# Hypertension Prevalence Among People Lifted Out of Poverty in China in 2018-2023: Retrospective Spatiotemporal Analysis

**DOI:** 10.2196/66501

**Published:** 2025-08-19

**Authors:** Ying Zhang, Dong Xia, Zhiyu Lv, Chennan Wu, Jiapeng Chen, Lulu Zhang

**Affiliations:** 1 School of Health Science and Engineering, University of Shanghai for Science and Technology, Shanghai, China; 2 Department of Military Health Management, College of Health Service, Naval Medical University, No. 41, Zhengtong Road, Wujiaochang Street, Yangpu DistrictShanghai, 200000, China, 86 81871421; 3 China Population and Development Research Center, Beijing, China

**Keywords:** hypertension, time series analysis, spatial analysis, trend surface analysis, public health, high blood pressure, blood pressure, China, Chinese, JoinPoint regression, temporal trends, spatial distribution, spatiotemporal analysis, global health, prevalence, disparities, poverty, non-communicable disease, epidemic, epidemiology

## Abstract

**Background:**

Hypertension is a significant global public health concern, with particular concern in China due to its widespread prevalence. The spatial distribution of hypertension varies significantly, revealing important regional disparities that may impact public health strategies and interventions.

**Objective:**

This study aimed to investigate the temporal trends and spatial distribution characteristics of hypertension prevalence among individuals lifted out of poverty in China, covering the period from 2018 to 2023.

**Methods:**

We used data from the National Health Poverty Alleviation Dynamic Management System to analyze hypertension prevalence among people lifted out of poverty from 2018 to 2023. Long-term trends were assessed using the Joinpoint regression model. Spatial distribution characteristics were examined through global and local spatial autocorrelation, cluster and outlier analyses, and trend surface analyses. These methods provided insights into the spatial aggregation and variability of hypertension prevalence across different regions.

**Results:**

From 2018 to 2023, the prevalence of hypertension among people lifted out of poverty in China increased from 2.71% to 7.29%. The highest rates were observed in the northeast, with Jilin Province ranking first for 3 consecutive years (2020‐2023), reaching 27.19% in 2023. Linxi County in Inner Mongolia had prevalence rates exceeding 40% for 5 years (2019‐2023), peaking at 47.99% in 2022. Among the 22 provinces containing poverty-stricken counties, 9 showed significant annual increases, with Guangxi having the highest annual percentage change at 27.9018% (95% CI 7.4095%‐52.3038%). Spatial analysis identified high-high clusters in northern provinces such as Hebei, Jilin, and Inner Mongolia, and low-low clusters in southwestern provinces such as Yunnan and Guizhou. Trend surface analysis revealed a distinct spatial gradient, with the northeast highest and the southwest lowest.

**Conclusions:**

The study revealed a generally increasing trend in hypertension prevalence among people lifted out of poverty in China from 2018 to 2023. The highest prevalence rates were concentrated in northeastern poverty-alleviated counties, while southwestern counties exhibited the lowest prevalence rates.

## Introduction

Hypertension is a global public health issue that poses a significant threat to human health and contributes substantially to the disease burden worldwide [1-3]. According to a global pooled analysis, the prevalence of hypertension has steadily increased over the past three decades, especially in low- and middle-income countries, including China [4]. In 2019, China had one of the highest numbers of people with hypertension globally, with substantial disparities across sex, region, and socioeconomic groups [5]. According to a nationwide survey of 298,438 individuals across 31 provinces conducted from 2020 to 2022, the prevalence among residents aged 18 years or older was 31.6%, with males (36.8%) having a higher prevalence than females (26.3%), and rural areas (33.7%) having a higher prevalence than urban areas (29.1%) [6]. Although prevention and control programs have expanded, the control rate of hypertension remains unsatisfactory in key populations [7,8]. While there is no cure for hypertension, medication can typically maintain blood pressure at a safe level in most patients, effectively reducing the prevalence and mortality rates of cardiovascular disease, stroke, and kidney disease [9,10]. However, structural disparities in health care access persist, particularly in under-resourced regions [11].

People lifted out of poverty are those who, through policy support and their own efforts, have succeeded in escaping poverty and reaching the minimum standard of living set by the state. According to China’s current poverty standards, those lifted out of poverty must meet the following conditions: an average annual per capita household income of more than 2300 yuan (at constant 2010 prices) and the realization of the principle of “two no worries, three guarantees,” which refers to not worrying about food or clothing, as well as guarantees for compulsory education, basic medical care, and housing security. Among this population, as an important target of precision poverty alleviation, the “established card households” are those poor families that have been included in the national poverty alleviation information system and dynamically managed throughout the poverty alleviation process. By the end of 2020, all 832 poverty-alleviated counties had met the criteria, resulting in a total of 98.99 million rural poor people being lifted out of poverty according to the current standards. This historic achievement significantly reduced absolute poverty in China and yielded profound social benefits. However, the health status of people in these counties still lags behind the national average. To address this, policies such as chronic disease subsidies, family doctor programs, and enhanced medical insurance have been implemented to promote equitable health care [12-14]. Nonetheless, research shows that individuals in poverty-alleviated counties remain disproportionately affected by chronic diseases, including hypertension [15].

Many scholars have studied the knowledge, treatment, and control of hypertension [16,17], but only a few have focused on hypertension prevalence among people lifted out of poverty. Despite the remarkable success of China’s efforts to combat poverty, these individuals still face certain vulnerabilities, especially in terms of their generally poor health status. Due to long-term poverty, they have experienced a relative lack of health management and medical resources, which has led to a higher prevalence of some chronic diseases. In this context, our research focused on analyzing hypertension prevalence among people lifted out of poverty, specifically examining its characteristics across temporal and spatial dimensions. This study aimed to provide a scientific basis for developing more targeted health interventions to further improve the quality of life and health of those who have been lifted out of poverty.

## Methods

### Data Sources

Hypertension prevalence data for people lifted out of poverty in the 832 poverty-alleviated counties nationwide were obtained from the National Health Poverty Alleviation Dynamic Management System. This system provides comprehensive information on people lifted out of poverty, including details on literacy levels, health status, family types, and ethnicity. Specific data include the number of patients, the number of people receiving assistance, the number of times assistance was provided, and reimbursement ratios. National county-level vector maps were obtained from China’s National Basic Geographic Information System.

### Descriptive Statistics

We described the temporal trends and regional distribution characteristics of hypertension prevalence among people lifted out of poverty from 2018 to 2023. Using the data on the number of patients and the number of people registered in each county, we analyzed the overall temporal trends in hypertension prevalence among this population nationwide. The 832 poverty-alleviated counties were spread across 22 provinces, and we conducted a statistical analysis of hypertension prevalence in the population lifted out of poverty in each province. In addition to the statistical analysis, we also analyzed the spatial distribution characteristics of hypertension prevalence in each county. Hypertension prevalence was calculated using the following formula:


$$Pi=\frac{Ni}{Ti}*100\%$$

$$Ppi=\frac{Npi}{Tpi}*100\%$$

$$Pci=\frac{Nci}{Tci}*100\%$$

Where *i* (2018≤*i*≤2023) denotes the six different years, *Pi* denotes the national prevalence of hypertension among people lifted out of poverty in each year, *Ni* denotes the number of people with hypertension among this population nationwide each year, and *Ti* denotes the total number of people lifted out of poverty nationwide each year. *p*(1≤*p*≤22) denotes the 22 provinces, *Ppi* denotes hypertension prevalence among people lifted out of poverty in each province each year, and *Npi* denotes the number of people with hypertension among people lifted out of poverty in each province each year. *Tpi* denotes the total number of people lifted out of poverty in each province each year. *c*(1≤*c*≤832) denotes the 832 counties, *Pci* denotes hypertension prevalence among people lifted out of poverty in each county each year, *Nci* denotes the number of patients with hypertension among those lifted out of poverty in each county each year, and *Tci* denotes the total number of people lifted out of poverty in each county each year.

### Time Series Analysis

We used the Joinpoint regression model [18] to analyze temporal trends in hypertension prevalence among people lifted out of poverty across 22 provinces from 2018 to 2023. The model detects statistically significant trend change points (inflection points) through segmented regression and calculates the annual percentage change (APC) for each period to assess how the trend is changing. In contrast to the autoregressive integrated moving average (ARIMA) model and exponential smoothing, Joinpoint allows data to show different trends over time.

The Joinpoint regression model relies on several key assumptions. First, it assumes that the error terms are independent and identically distributed. To address potential autocorrelation in the data, we examined the residuals for any signs of serial correlation and applied appropriate adjustments if necessary. Second, the model assumes homoscedasticity, meaning that the variance of the error terms is constant over time. In cases where heteroscedasticity was detected, we used weighted least squares to account for varying error variances. Finally, the model assumes that the trend changes are continuous at the joinpoints, ensuring smooth transitions between different linear segments. For our study, the log-transformed independent variable for prevalence was time (year), and the dependent variable was prevalence. We used the observation series (*i_1_*, *Ppi_1_*), ...(*i_n_, Ppi_n_*), (*i_1_*≤...≤*i_n_*), where *n* is the study period, and the regression equation for the log-linear model is:


$$E\left[Ppi|i\right]=e^{\beta_{0}+\beta_{1}i+\delta_{1}{\left(i-\tau_{1}\right)}^{+}+\ldots +\delta_{k}{\left(i-\tau_{k}\right)}^{+}}$$

where *e* is the base of the natural logarithm, *k* denotes the number of turning points, *β_0_* is the intercept parameter, *β_1_* is the regression coefficient for the trend, and *δk* denotes the regression coefficient for segment *k*. When (*i-τ_k_*)>0, then (*i-τ_k_*)^+^)=*i-τ_k_*; otherwise, (*i-τ_k_*_)_^+^)=0.

We used the APC as well as the 95% CI to describe the change in each line segment, the change between inflection points, and the change over the entire period. The formula used is as follows:


$$APC=\left(e^{\beta_{1}}-1\right)\times 100\%$$

### Spatial Analysis

In our study, we used both global and local spatial autocorrelation analyses to explore the spatial distribution characteristics of hypertension prevalence among people lifted out of poverty. For global spatial autocorrelation, we applied Moran I [19] to assess the spatial correlation between data points across the study area. Moran I ranges from −1 to 1. Positive values indicate positive autocorrelation (spatial clustering); negative values indicate negative autocorrelation (spatial dispersion); and a value of 0 indicates no spatial autocorrelation. The formula for calculating Moran I is as follows:


$$I=\frac{c}{W}\cdot \frac{\sum_{k=1}^{c}\;\sum_{j=1}^{c}\;w_{kj}\left(Pc_{k}-\overset{-}{Pc}\right)\left(Pc_{j}-\overset{-}{Pc}\right)}{\sum_{k=1}^{c}\;{\left(Pc_{k}-\overset{-}{Pc}\right)}^{2}}$$

where *c* is the sample size, that is, the number of counties in the study area, *W* is the sum of the spatial weight matrix, that is, $W=\sum_{k=1}^{c}\;\sum_{j=1}^{c}\;w_{kj}$*, Pc_k_*, and *Pc_j_* are the prevalence rates of hypertension in the *k*th and *j*th counties, respectively, and $\overset{-}{Pc}$ is the mean of the prevalence rates of hypertension in counties that have escaped poverty. *w_kj_* is an element in the spatial weight matrix, which denotes the spatial relationship between *k*th and *j*th counties (which can usually be a neighborhood weight or a distance weight).

In this study, we used the method of clustering and outlier analysis (Anselin Local Moran I) to examine the local spatial autocorrelation, which can effectively identify local autocorrelation patterns in spatial data and help reveal intrinsic patterns and anomalous features of spatial phenomena. Based on the calculated Local Moran I and the results of the significance tests, four types of spatial clusters and outliers, namely high-high, low-low, high-low, and low-high, were identified. The formula for clustering and outlier analysis is as follows:


$$I_{k}=\frac{\left(Pc_{k}-\overset{-}{Pc}\right)}{m_{2}}\sum_{j=1}^{c}\;w_{kj}\left(Pc_{j}-\overset{-}{Pc}\right)$$

$$m_{2}=\frac{1}{c}\sum_{k}\;{\left(Pc_{k}-\overset{-}{Pc}\right)}^{2}$$

where *I_k_* is the local Moran I value for the *k*th county and *m2* is the variance of the prevalence rate across the 832 poverty-alleviated counties.

In spatial analysis, *z* values and *P* values are two key indicators used to assess statistical significance. The *z* value represents the standardized value of Moran I and measures the deviation of the observed value from the expected value. A larger (positive) *z* value indicates that clusters of higher values are more significant, while a smaller (negative) *z* value indicates that clusters of lower values are more significant. The *P* values indicate the probability of the observed outcome occurring by chance, reflecting the statistical significance of the result.

### Trend Surface Analysis

Trend surface analysis [20] is a spatial data analysis technique used to describe the overall trend in spatial data by fitting a mathematical model. We used a second-order polynomial trend surface model to analyze the spatial distribution of hypertension prevalence among people lifted out of poverty. The coefficient of determination (*R*²) of the model was 0.569, and the root-mean-square error was 3.541, indicating that the model could reveal the spatial trend better, but there is still room for improvement. Comparing with first-order and higher-order polynomials, the first-order model was unable to capture nonlinear variations, and the higher-order model was prone to overfitting and did not significantly improve the goodness-of-fit. We used the geographic coordinates (longitude and latitude) of each province as the independent variables (x-axis and y-axis) and the prevalence rate as the dependent variable (z-axis). In this setup, the x-axis increases from left to right, representing longitude from low to high (west to east); the y-axis increases from bottom to top, representing latitude from low to high (south to north); and the z-axis increases from low to high, indicating higher prevalence rates. In the 3D spatial trend surface analysis graphs, the xy plane indicates the distribution of people lifted out of poverty across different latitude and longitude locations. The xz plane illustrates the trend of hypertension prevalence relative to longitude, and the yz plane indicates the trend of hypertension prevalence relative to latitude.

### Ethical Considerations

The original data were collected through a national platform established and managed by the National Health Commission of China. In accordance with national regulations, data collection activities conducted under the Commission’s authority are subject to institutional review board (IRB) approval. This study involved a secondary analysis of deidentified, aggregate county-level data, with no individual identifiers or biospecimens, and therefore did not require additional IRB review. According to International and National Ethical Guidelines (eg, 45 CFR 46.104[d] [4] and Article 23 of China’s Ethical Review Guidelines), such secondary analyses of anonymized data are exempt from further IRB approval. The authors have obtained formal permission to access and use the data for academic research purposes.

## Results

### Epidemiological Characteristics

**Figure 1.**
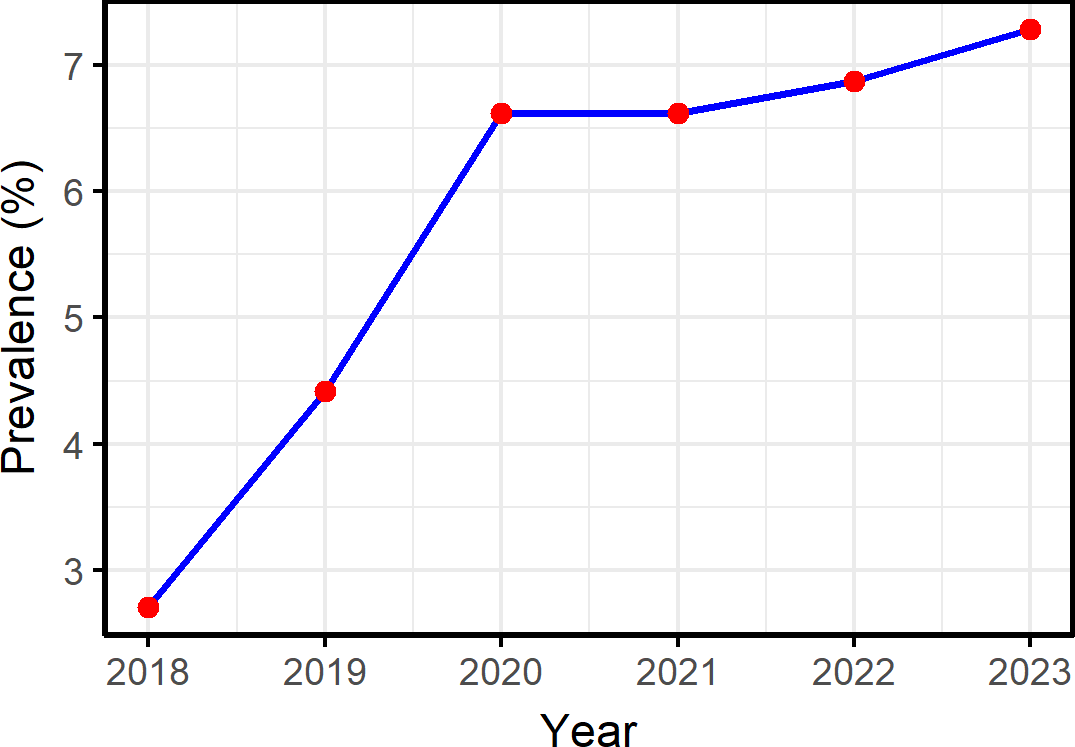
Temporal trends in hypertension prevalence among people lifted out of poverty in China (2018‐2023).

**Figure 2.**
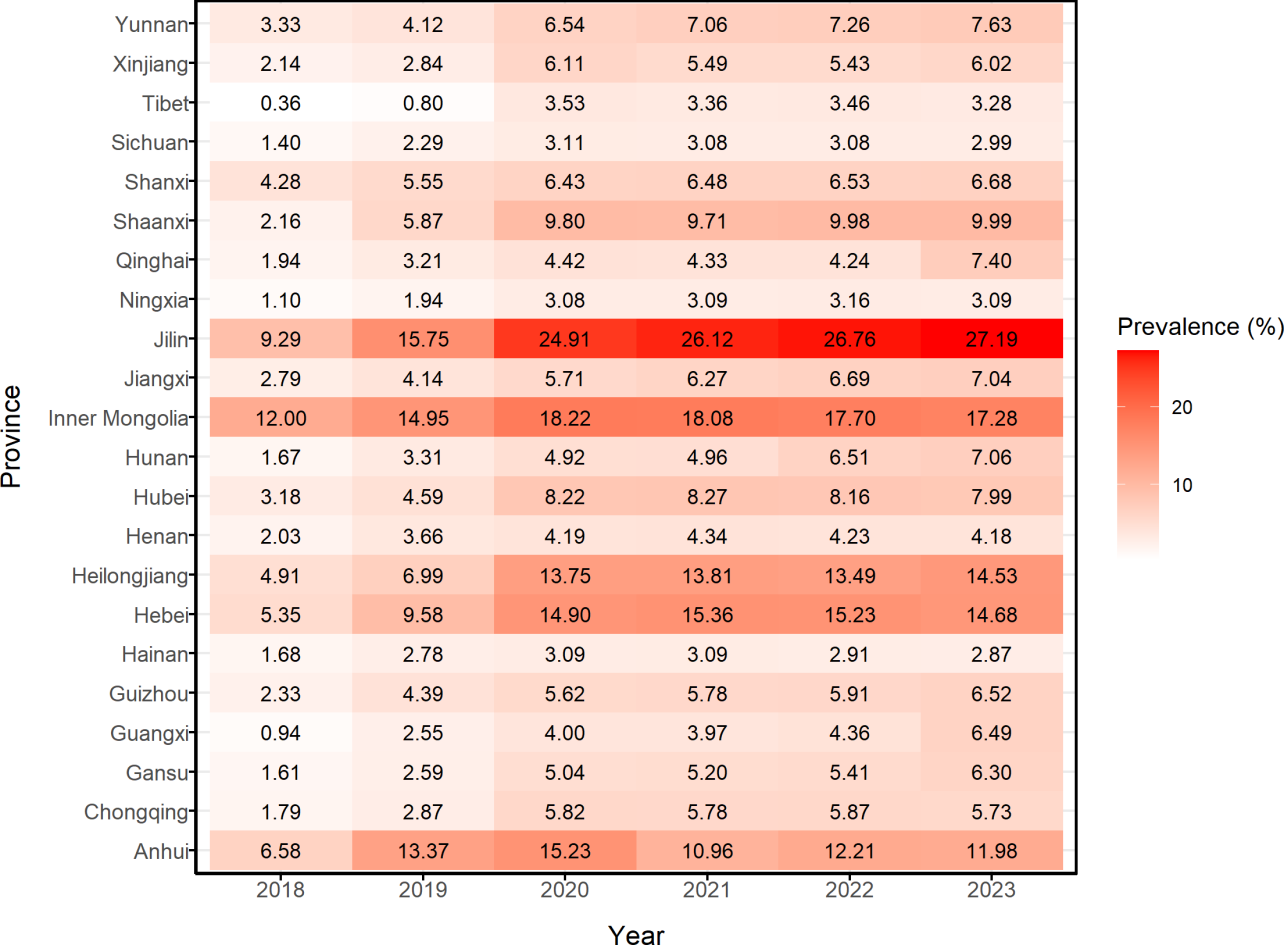
Heatmap of hypertension prevalence among the people lifted out of poverty in 22 provinces of China (2018‐2023).

**Figure 3.**
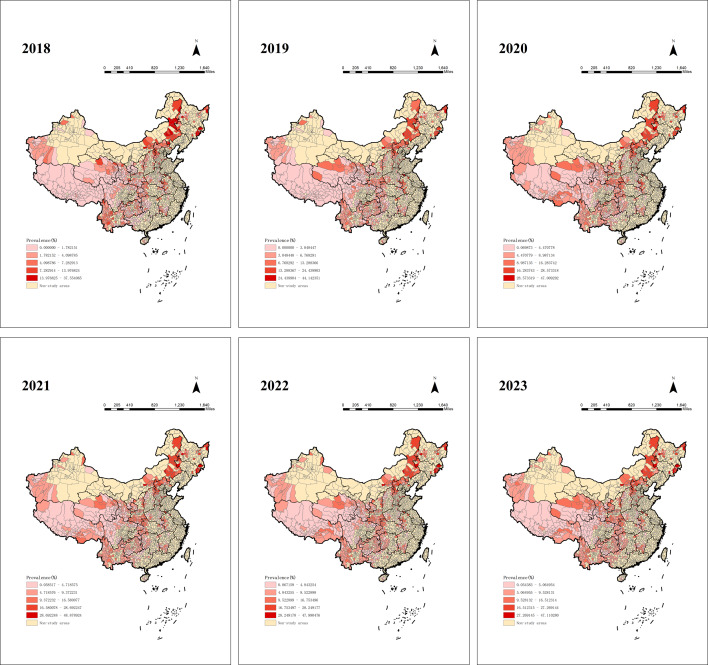
County-level hypertension prevalence among poverty-alleviated populations in China (2018‐2023).

**Figure 4.**
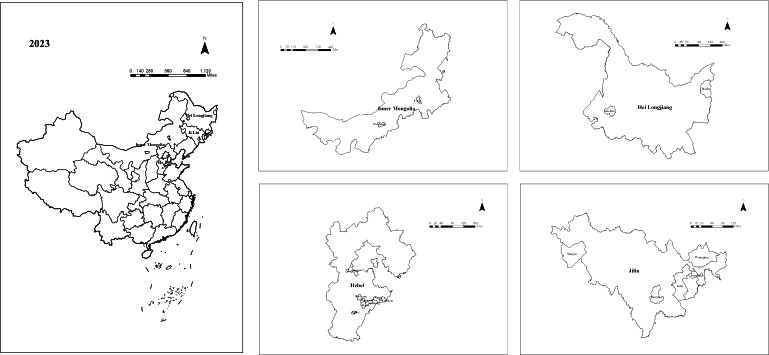
Geographic distribution of the 17 counties with the highest hypertension prevalence among people lifted out of poverty in China (2023).

Between 2018 and 2023, hypertension prevalence among people lifted out of poverty showed an overall increasing trend. As shown in Figure 1, the most significant rise occurred between 2018 and 2020, with the prevalence increasing from approximately 2.71% in 2018 to approximately 6.62% in 2020. There was a slight decrease in prevalence from 2020 to 2021, but the overall trend remained relatively stable. From 2021 onward, the prevalence rate began to rise again gradually, reaching a 6-year peak of approximately 7.29% in 2023. We analyzed the annual trend of hypertension prevalence among people lifted out of poverty across the 22 provinces containing poverty-alleviated counties. As shown in Figure 2, hypertension prevalence varied across provinces from 2018 to 2023. Four provinces—Jilin, Heilongjiang, Inner Mongolia Autonomous Region, and Hebei—exhibited the highest prevalence rates. Notably, Jilin Province had the highest prevalence across all 6 years, peaking at approximately 27.19% in 2023. In addition, we analyzed hypertension prevalence in each of the 832 poverty-alleviated counties. As shown in Figure 3, most counties with high hypertension prevalence among people lifted out of poverty were located in the northeast, followed by the northwest and southwest regions, with consistent regional distribution over the 6 years. Analysis revealed that in 2023, the 17 counties with the highest prevalence were distributed across four regions: Jilin Province (n=5), Heilongjiang Province (n=2), Inner Mongolia Autonomous Region (n=2), and Hebei Province (n=8), as illustrated in Figure 4. Notably, Linxi County in the Inner Mongolia Autonomous Region had a prevalence rate exceeding 40% from 2019 to 2023, peaking at 47.99% in 2022. In parallel, Longjing City in Jilin Province showed a continuous increase in prevalence, with an average annual increase of about 3%.

### Temporal Distribution

**Figure 5.**
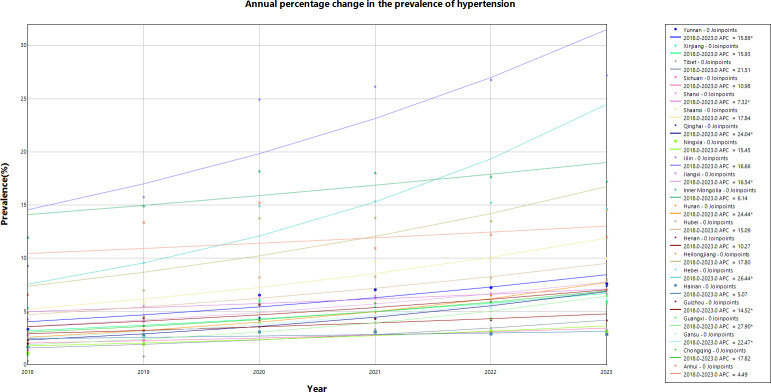
Annual changes in hypertension prevalence among people lifted out of poverty in 22 provinces in China (2018-2023).

We analyzed the time trend of hypertension prevalence among people lifted out of poverty across 22 provinces, as shown in Figure 5. Statistically significant APC values were found in only 9 provinces: Yunnan, Shanxi, Qinghai, Jiangxi, Hunan, Hebei, Guizhou, Guangxi, and Gansu. In these provinces, hypertension prevalence showed a consistent year-by-year increase. Guangxi Province had the highest APC value at 27.9018% (95% CI 7.4095%‐52.3038%), followed by Hebei Province with an APC value of 26.4411% (95% CI 0.5839%‐58.9456%), and Hunan Province with the third highest APC value of 24.4372% (95% CI 9.1285%‐41.8933%), as shown in Table 1.

**Table 1.** Annual trends in hypertension prevalence among people lifted out of poverty in 22 provinces in China (2018-2023).

Cohort	APC^a^ (95% CI)	*t* test (*df*)	*P* value
Yunnan	15.8795^b^ (3.5653 to 29.6579)	3.6422 (5)	.02
Xinjiang	15.9303 (−5.1830 to 41.7450)	2.0414 (5)	.11
Tibet	21.5128 (−20.1508 to 84.9156)	1.2884 (5)	.27
Sichuan	10.9760 (−3.9215 to 28.1833)	2.0059 (5)	.12
Shanxi	7.3244^b^ (0.2287 to 14.9223)	2.8692 (5)	.05
Shaanxi	17.8446 (−7.3675 to 49.9187)	1.8938 (5)	.13
Qinghai	24.0424^b^ (9.1032 to 41.0272)	4.6614 (5)	.01
Ningxia	15.4480 (−3.4627 to 38.0631)	2.2295 (5)	.09
Jilin	16.6599 (−1.0574 to 37.5497)	2.5973 (5)	.06
Jiangxi	16.5443^b^ (5.2907 to 29.0006)	4.1861 (5)	.01
Inner Mongolia	6.1368 (−2.2056 to 15.1910)	2.0200 (5)	.11
Hunan	24.4372^b^ (9.1285 to 41.8933)	4.6240 (5)	.01
Hubei	15.0930 (−4.3224 to 38.4482)	2.1124 (5)	.10
Henan	10.2661 (−4.3873 to 27.1653)	1.9029 (5)	.13
Heilongjiang	17.7968 (−2.8046 to 42.7649)	2.3656 (5)	.08
Hebei	26.4411^b^ (0.5839 to 58.9456)	2.8471 (5)	.05
Hainan	5.0657 (−6.8447 to 18.4990)	1.1403 (5)	.32
Guizhou	14.5227^b^ (0.2244 to 30.8609)	2.8231 (5)	.05
Guangxi	27.9018^b^ (7.4095 to 52.3038)	3.9130 (5)	.02
Gansu	22.4657^b^ (2.7094 to 46.0222)	3.1983 (5)	.03
Chongqing	17.8208 (−5.5987 to 47.0505)	2.0546 (5)	.11
Anhui	4.4920 (−13.2676 to 25.8881)	0.6549 (5)	.55

a APC: annual percentage change.

b Indicates that the annual percent change is significantly different from zero at the α=.05 level.

### Spatial Distribution

**Figure 6.**
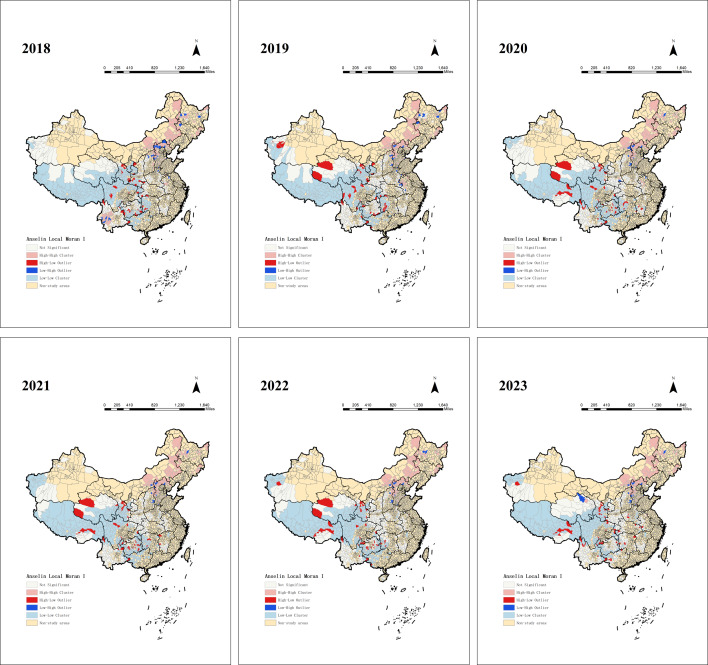
Local spatial clustering of hypertension prevalence among poverty-alleviated counties in China (2018-2023).

As shown in Table 2, the global spatial autocorrelation analysis revealed that from 2018 to 2023, both Moran I and *z* values were positive. This indicates a significant positive spatial autocorrelation in the distribution of hypertension prevalence among people lifted out of poverty at the county level, reflecting notable spatial clustering. Except for 2020, the variance in the remaining years was 0.000073. This small variance suggests that Moran I showed minimal volatility, indicating stable spatial autocorrelation and limited spatial change trends. As shown in Figure 6, we performed clustering and outlier analysis on hypertension prevalence among people lifted out of poverty in the 832 poverty-alleviated counties. The results showed that the spatial distribution of hypertension prevalence from 2018 to 2023 presents four types of clustering. The high-high clustered areas were mainly distributed in Hebei, Shanxi, Inner Mongolia Autonomous Region, Jilin, Heilongjiang, and Anhui Provinces, where hypertension prevalence was significantly higher than that of the neighboring regions and showed persistent clustering, which may reflect a heavier disease burden in those areas. The identification of high-high aggregation areas is crucial for developing targeted disease prevention and control strategies; therefore, we further identified a county-level list of high-high aggregation areas with high prevalence of hypertension in 2018‐2023 (refer to Multimedia Appendix 1 for details). The low-low aggregation areas were concentrated in Hunan, Guangxi Zhuang Autonomous Region, Chongqing, Sichuan, Guizhou, Yunnan, Tibet Autonomous Region, Shaanxi, Gansu, Qinghai, Ningxia Hui Autonomous Region, and Xinjiang Uygur Autonomous Region, where hypertension prevalence was significantly lower than that of neighboring regions. High-low aggregation areas were mainly distributed in Hunan, Sichuan, Yunnan, Guizhou, and Gansu Provinces, where hypertension prevalence was relatively high, while neighboring regions had lower rates, possibly reflecting localized disease hotspots. Low-high clusters were mainly located in Hebei, Shanxi, and Heilongjiang Provinces, where hypertension prevalence was lower in these regions but higher in neighboring areas, possibly representing a transitional area of disease spread.

**Table 2.** Global spatial autocorrelation analysis of hypertension prevalence among people lifted out of poverty in China (2018-2023).

Year	Moran I	Expected I	Variance	Z value	*P* value	Result
2018	0.256240	−0.001215	0.000073	30.189126	<.001	Clustered
2019	0.272386	−0.001215	0.000073	31.981011	<.001	Clustered
2020	0.319228	−0.001215	0.000074	37.365603	<.001	Clustered
2021	0.329789	−0.001215	0.000073	38.626703	<.001	Clustered
2022	0.315589	−0.001215	0.000073	36.961519	<.001	Clustered
2023	0.282911	−0.001215	0.000073	33.155462	<.001	Clustered

### Trend Surface Analysis

**Figure 7.**
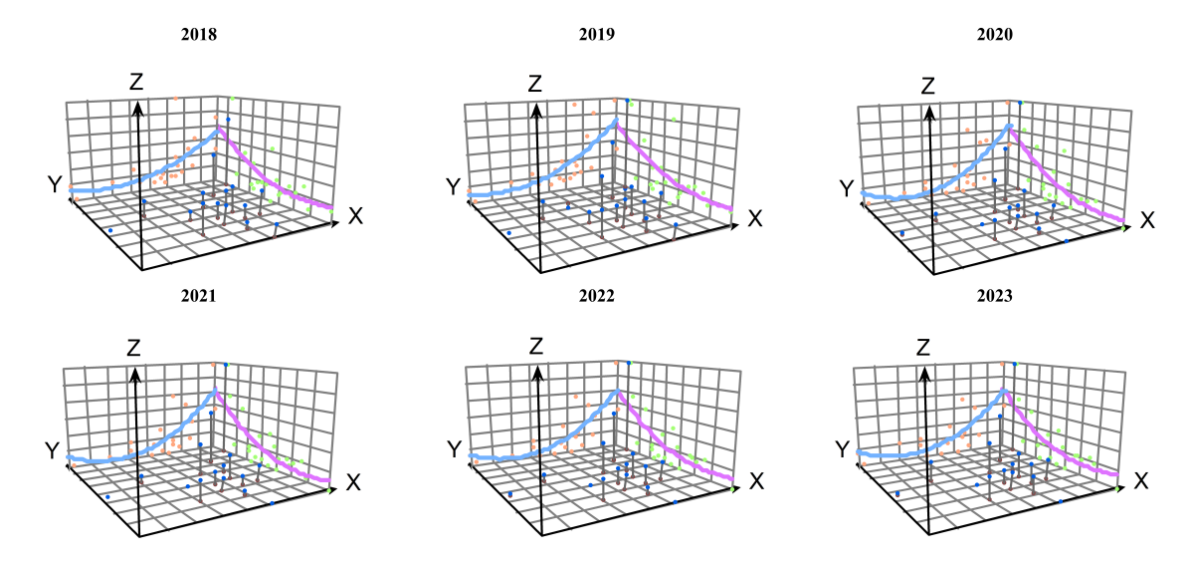
Trend surface analysis of hypertension prevalence among people lifted out of poverty in 22 provinces in China (2018‐2023).

As shown in Figure 7, there was a clear spatial heterogeneity in hypertension prevalence among people lifted out of poverty at the provincial level. From 2018 to 2023, hypertension prevalence initially decreased with increasing longitude but then increased again at a faster rate. This indicates that the eastern part of the country generally had a higher prevalence than the western part, with the prevalence being lowest in the central and western regions. In 2018, 2021, and 2023, hypertension prevalence among people lifted out of poverty first decreased and then increased with increasing latitude. In contrast, in the remaining years, the prevalence increased with latitude. Overall, the northern regions exhibited significantly higher prevalence rates than the southern, with the southern and south-central provinces having the lowest prevalence. Therefore, we conclude that the northeastern region generally has the highest prevalence of hypertension among people lifted out of poverty.

## Discussion

### Principal Findings

From 1990 to 2019, the number of people with hypertension doubled globally [4]. In our study, hypertension prevalence among people lifted out of poverty in China increased consistently from 2018 to 2023, with a rise of approximately 4.59% in 2023 compared to the level in 2018. The APC values for all provinces were also positive, indicating a general upward trend. This increase in hypertension prevalence in China is likely attributable to rising life expectancy and lifestyle changes [21]. There are four potential factors contributing to the overall increase in hypertension prevalence. First, the Scientific Research Report on Dietary Guidelines for Chinese Residents*,* issued in 2021, highlights changes in dietary structure. Specifically, there has been an increase in the consumption of high-calorie, high-fat, high-sugar, and high-salt foods. In addition, the rates of overweight and obesity in rural areas have risen more significantly than in urban areas. Obesity is a major risk factor for hypertension [22]. Second, inadequate sleep and poor sleep quality are associated with an increased risk of hypertension [23]. Insufficient sleep can lead to cognitive decline and poor mental health, while high-pressure environments, mental overstimulation, and anxiety can trigger hypertension [24,25]. Third, population aging significantly contributes to the rising prevalence of hypertension. As Chinese society ages, the proportion of older individuals is increasing, and this age group is particularly susceptible to chronic diseases, such as hypertension [26]. Fourth, the increased prevalence of hypertension is not only closely related to environmental factors and lifestyle but is also significantly influenced by hereditary factors. Studies have shown that individuals with a family history of hypertension have a significantly increased risk of developing the condition. This is because certain genetic variants and genes can affect blood pressure regulatory mechanisms, making individuals with genetic susceptibility more prone to developing hypertension [27,28].

According to the spatial autocorrelation analysis, the high-high aggregation area is mainly distributed in the three northeastern provinces and northern China. The low-high aggregation area is distributed at the junction of northern China and northeastern China, and the high-low aggregation and low-low aggregation areas are mainly located in the west. Trend surface analysis shows that hypertension prevalence is higher in the east than in the west, and in the north than in the south. As early as 1994, a study found that hypertension prevalence was higher in northern China than in southern regions [29].

The spatial heterogeneity in hypertension prevalence among people lifted out of poverty may be explained by the following three factors: First, some studies have shown that high-altitude areas may experience abnormal blood pressure regulation due to hypoxia, which can increase the risk of hypertension [30-32]. The lower prevalence of hypertension in Tibet and Qinghai Provinces may be because residents have been exposed to low oxygen levels from birth and have developed adaptations to better cope with high-altitude conditions [33]. In addition, the diversity of physiological changes may result in different adaptations among high-altitude populations. For instance, research has shown that Tibetan populations exhibit better adaptation than non-Tibetan populations due to higher capillary density and greater oxygen delivery [34]. Experimental studies have also indicated that in Tibetan populations, hypertension prevalence increases by 1.2% for every 100-meter increase in altitude, a phenomenon not observed in all high-altitude populations [35]. In non-Tibetan populations, there is no significant correlation between increased altitude and changes in blood pressure [36]. Second, temperature changes can also impact blood pressure. A study conducted in northwest China found a significant negative correlation between outdoor temperature and blood pressure, with blood pressure demonstrating notable seasonal variations [37]. This seasonal variation in blood pressure is likely due to changes in ambient temperature, as another study showed that the seasonal variation disappeared after adjusting for temperature [38]. Larger differences in blood pressure between colder and warmer months may increase hypertension prevalence, potentially elevating cardiovascular risk, especially among individuals at high cardiovascular risk and older individuals [39]. Third, dietary differences between the northern and southern regions of China also play a significant role. Regular consumption of high-sodium diets, energy-dense foods, fats, refined carbohydrates, added sugars, and low amounts of fruits and vegetables increases the risk of hypertension and cardiovascular disease. According to the Chinese Guidelines for Adults with Hypertension, issued in 2023, the diet in northeastern China is characterized by stews with a heavy taste, while in central China, food processing methods, such as pickling and curing, are popular, also resulting in heavily seasoned dishes. Meanwhile, southwestern China is known for its preference for spicy food. These guidelines emphasize the need to control oil and salt use across all regions. The Dietary Approaches to Stop Hypertension (DASH) diet was introduced in the United States in 1997 as a dietary pattern to prevent and manage hypertension. This diet focuses on reducing fats and oils while ensuring adequate intake of vegetables, fruits, and low-fat dairy products to maintain optimal levels of potassium, magnesium, and calcium, all of which significantly reduce blood pressure [40-42]. In addition, the Chinese heart-healthy diet and its Sichuan cuisine version, tailored for the Chinese population, have shown significant positive effects in reducing the risk of developing hypertension [43,44].

High-high aggregation areas should be used as key intervention areas to strengthen screening and early intervention, optimize medical resource allocation, and promote low-salt and low-fat diets and moderate exercise through health education. Although the current burden of disease in low-low aggregation areas is low, continuous monitoring is needed. High-low aggregation areas may reflect localized disease hotspots, requiring special attention to the distribution of health care resources, increased investment in primary care, and the development of personalized prevention and control strategies. Low-high aggregation areas may be transitional areas for the spread of the disease, and monitoring and regional joint prevention and control should be strengthened to prevent the spread of hypertension from areas of high prevalence to areas of low prevalence.

Uncontrolled hypertension can result in severe health issues, including stroke, cardiovascular diseases, and kidney diseases, leading to unnecessary human burden and premature death. Despite significant investments by the state in poverty alleviation and rural revitalization [45], the health service system remains inadequate. The prevention and control of chronic diseases, such as hypertension, still face considerable challenges due to limited medical resources and a lack of widespread health education [46]. Both national and local governments should enhance interregional collaborative efforts to address the regional differences and clustering patterns in hypertension prevalence [47]. Establishing cross-regional collaboration mechanisms for hypertension prevention and control, information sharing, resource complementation, and joint efforts can enhance the overall effectiveness of these initiatives. In addition, increased investment in medical resources in impoverished areas has optimized the allocation of these resources. The capacity to prevent and control hypertension in these regions has been strengthened through the development of primary health care institutions, training of primary health care personnel, and the promotion of telemedicine. Nurses can make significant contributions to national hypertension control efforts by leveraging evidence-based practices for the prevention, diagnosis, treatment, and management of hypertension [48]. Furthermore, strengthening health education and public awareness initiatives is crucial for promoting healthy lifestyles and dietary habits. Raising public awareness about hypertension and its prevention involves disseminating knowledge through health promotion activities and educational interventions. Providing targeted education to patients with hypertension can encourage the adoption of healthy behaviors and improve overall health outcomes [49].

### Limitations

There are some limitations to this study. First, using the total recorded population in each county that has escaped poverty as the county’s total population may introduce statistical errors, as this figure may include mobile populations. Second, the study did not account for seasonal changes, which affect both temperature and dietary patterns and can influence hypertension prevalence. Finally, while this study analyzed the spatial and temporal prevalence of hypertension, further research is needed to examine the underlying factors affecting hypertension prevalence to develop more targeted and effective prevention guidelines.

### Conclusions

In this study, we determined the temporal trends and spatial distribution of hypertension prevalence among Chinese individuals lifted out of poverty from 2018 to 2023. Our findings reveal an overall increasing trend in hypertension prevalence year by year. High-prevalence areas were notably concentrated in the northeastern poverty-alleviated counties, while low-prevalence areas were found primarily in the southwestern counties.

## Supplementary material

10.2196/66501 Multimedia Appendix 1 List of counties with high concentrations of hypertension prevalence (2018-2023).

## Abbreviations

APC: annual percentage change
ARIMA: autoregressive integrated moving average
DASH: Dietary Approaches to Stop Hypertension
IRB: institutional review board
