# Supplementary material for: Hypertension Prevalence Among People Lifted Out of Poverty in China in 2018-2023: Retrospective Spatiotemporal Analysis
Source: JMIR Public Health Surveill. 2025 Aug 19;11:e66501. doi: 10.2196/66501 (PMC12364423; doi:10.2196/66501)
Supplement: Multimedia Appendix 1 [file publichealth-v11-e66501-s001.docx]

**2018:**

Lingshou County, Zanhuang County, Qinglong Manchu Autonomous County, Daming County, Lincheng County, Julu County, Xinhe County, Guangzong County, Pingxiang County, Wei County, Laishui County, Fuping County, Laiyuan County, Wangdu County, Yi County, Quyang County, Shunping County, Xuanhua District, Wanquan District, Kangbao County, Guyuan County, Yu County, Yangyuan County, Huai’an County, Chengde County, Longhua County, Fengning Manchu Autonomous County, Pingquan County, Yanshan County , Nanpi County, Wuyi County, Wuqiang County, Raoyang County, Fucheng County, Yunzhou District, Yanggao County, Tianzhen County, Guangling County, Lingqiu County, Hunyuan County, Pingshun County, Huguan County, Wuxiang County, Youyu County, Zuoquan County, Heshun County, Wutai County, Pianguan County, Wuchuan County, Ar Horqin Banner, Bahrain Left Banner, Bahrain Right Banner, Linxi County, Wengniute Banner, Harqin Banner, Ningcheng County, Aohan Banner, Khorqin Left Wing Middle Banner, Khorqin Left Wing Rear Banner, Kulun Banner, Naiman Banner, Morin Dawa Daur Autonomous Banner, Oroqen Autonomous Banner, Zhuozi County, Huade County, Shangdu County, Xinghe County, Chahar Right wing Front banner, Chahar Right wing Middle banner, Chahar Right wing Rear banner, Siziwang Flag, Khorqin Right Wing Front banner, Khorqin Right Wing Middle banner, Jalaid Banner, Tuquan County, Sonid Right Banner, Taibus Banner, Zhengxiangbai Banner, Jingyu County, Zhenlai County, Tongyu County, Longjing City, Helong City, Wangqing County, Antu County, Yanshou County, Longjiang County, Tailai County, Gannan County, Suibin County, Lindian County, Tangyuan County, Tongjiang City, Fuyuan City, Wangkui County, Qinggang County, Mingshui County, Hailun City, Shou County, Yingdong District, Xiao County, Huoqiu County, Fan County, Taiqian County, Longyang District, Shidian County, Lvning County County, Jinggu Dai and Yi Autonomous County, Zhenyuan Yi Hani Lahu Autonomous County, Linxiang District, Yun County, Yongde County, Zhenkang County, Shuangjiang Lahu Va Blang Dai Autonomous County, Shuangbai County, Mouding County, Nanhua County, Yao 'an County, Dayao County, Yangbi Yi Autonomous County, Xiangyun County, Binchuan County, Midu County, Nandong Yi Autonomous County, Weishan Yi Hui Autonomous County, Yongping County, Arshan City

**2019:**

Lingshou County, Zanhuang County, Qinglong Manchu Autonomous County, Daming County, Lincheng County, Julu County, Xinhe County, Guangzong County, Pingxiang County, Wei County, Laishui County, Fuping County, Tang County, Laiyuan County, Wangdu County, Yi County, Quyang County, Shunping County, Xuanhua District, Wanquan District, Chongli District, Kangbao County, Guyuan County, Shangyi County, Yu County, Yangyuan County, Huai’an County, Chicheng County, Chengde County, Longhua County, Fengning Manchu Autonomous County, Weichang Manchu Mongolian Autonomous County, Pingquan City, Yanshan County, Nanpi County, Wuyi County, Wuqiang County, Raoyang County, Fucheng County, Yunzhou District, Yanggao County, Tianzhen County, Guangling County, Lingqiu County, Hunyuan County, Pingshun County, Huguan County, Youyu County, Heshun County, Wutai County, Wuchuan County, Ar Horqin Banner, Bahrain Left Banner, Bahrain Right Banner, Linxi County, Wengniute Banner , Harqin Banner, Ningcheng County, Aohan Banner, Khorqin Left Wing Middle Banner, Khorqin Left Wing Rear Banner, Kulun Banner, Naiman Banner, Morin Dawa Daur Autonomous Banner, Oroqen Autonomous Banner, Zhuozi County, Huade County, Shangdu County, Xinghe County, Chahar Right wing Front banner, Chahar Right wing Middle banner, Chahar Right wing Rear banner, Siziwang Banner, Arshan City, Khorqin Right Wing Front banner, Khorqin Right Wing Middle banner, Jalaid Banner, Tuquan County, Sonid Right Banner, Taibus Banner, Zhengxiangbai Banner, Jingyu County, Tongyu County, Da 'an City, Longjing City, Helong City, Wangqing County, Antu County, Longjiang County, Tailai County, Gannan County, Raohe County, Huachuan County, Tangyuan County, Tongjiang City, Fuyuan City, Qinggang County, Shou County, Yingdong District, Linquan County, Funan County, Yingshang County, Dangshan County, Xiao County, Lingbi County County, Sixian County, Yu 'an District, Huoqiu County, Shucheng County, Lixin County, Fan County, Taiqian County, Huaibin County

**2020:**

Lingshou County, Zanhuang County, Pingshan County, Qinglong Manchu Autonomous County, Daming County, Lincheng County, Julu County, Xinhe County, Guangzong County, Pingxiang County, Wei County, Laishui County, Fuping County, Tang County, Laiyuan County, Wangdu County, Yi County, Quyang County, Shunping County, Xuanhua District, Wanquan District, Chongli District, Kangbao County, Guyuan County, Shangyi County, Yu County, Yangyuan County, Huai 'an County, Chicheng County, Chengde County, Longhua County, Fengning Manchu Autonomous County, Weichang Manchu Mongolian Autonomous County, Pingquan City, Yanshan County, Nanpi County, Wuyi County, Wuqiang County, Raoyang County, Fucheng County, Yunzhou District, Yanggao County, Guangling County, Lingqiu County, Hunyuan County, Youyu County, Wutai County, Fanshi County, Wuchuan County, Ar Horqin Banner, Bairin left banner, Bairin right banner, Linxi County, Wengniute Banner, Harqin Banner, Ningcheng County, Aohan Banner, Khorqin Left Wing Middle Banner, Khorqin Left Wing Rear Banner, Kulun Banner, Naiman Banner, Morin Dawa Daur Autonomous Banner, Oroqen Autonomous Banner, Zhuozi County, Huad County, Shangdu County, Chahar Right wing Middle banner, Chahar Right wing Rear banner, Siziwang Banner, Arshan City, Khorqin Right Wing Front banner, Khorqin Right Wing Middle banner, Jalaid Banner, Tuquan County, Sonid Right Banner, Taibus Banner, Zhengxiangbai Banner, Jingyu County, Zhenlai County, Tongyu County, Da 'an City, Longjing City, Helong City, Wangqing County, Antu County, Yanshou County, Longjiang County, Tailai County, Gannan County, Fuyu County, Kedong County, Baiquan County, Suibin County, Raohe County, Lindian County, Huannan County, Huachuan County, Tangyuan County, Tongjiang City, Fuyuan City, Wangkui County, Lanxi County, Qinggang County, Mingshui County, Shou County, Yingdong District, Yingshang County, Xiao County, Yu 'an District, Huoqiu County, Lixin County, Fan County, Taiqian County

**2021:**

Lingshou County, Zanhuang County, Pingshan County, Qinglong Manchu Autonomous County, Daming County, Lincheng County, Julu County, Xinhe County, Guangzong County, Pingxiang County, Wei County, Laishui County, Fuping County, Tang County, Laiyuan County, Wangdu County, Yi County, Quyang County, Shunping County, Xuanhua District, Wanquan District, Chongli District, Kangbao County, Guyuan County, Shangyi County, Yu County, Yangyuan County, Huai 'an County, Chicheng County, Chengde County, Longhua County, Fengning Manchu Autonomous County, Weichang Manchu Mongolian Autonomous County, Pingquan City, Yanshan County, Nanpi County, Wuyi County, Wuqiang County, Raoyang County, Fucheng County, Yunzhou District, Yanggao County, Guangling County, Lingqiu County, Hunyuan County, Youyu County, Fanshi County, Wuchuan County, Ar Horqin Banner, Bahrain left banner, Bahrain right banner, Linxi County, Wengniute Banner, Harqin Banner, Ningcheng County, Aohan Banner, Khorqin Left Wing Middle Banner, Khorqin Left Wing Rear Banner, Kulun Banner, Naiman Banner, Morin Dawa Daur Autonomous Banner, Oroqen Autonomous Banner, Zhuozi County, Huade County, Shangdu County, Xinghe County, Chahar Right wing Middle banner, Chahar Right wing Rear banner, Siziwang Flag, Arshan City, Khorqin Right Wing Front banner, Khorqin Right Wing Middle banner, Jalaid Banner, Tuquan County, Sonid Right Banner, Taibus Banner, Zhengxiangbai Banner, Jingyu County, Zhenlai County, Tongyu County, Da 'an City, Longjing City, Helong City, Wangqing County, Antu County, Yanshou County, Longjiang County, Tailai County, Gannan County, Fuyu County, Kedong County, Baiquan County, Suibin County, Raohe County, Lindian County, Huannan County, Huachuan County, Tangyuan County, Tongjiang City, Fuyuan City, Wangkui County, Lanxi County, Qinggang County, Mingshui County, Fan County, Taiqian County

**2022:**

Lingshou County, Zanhuang County, Pingshan County, Qinglong Manchu Autonomous County, Daming County, Lincheng County, Julu County, Xinhe County, Guangzong County, Pingxiang County, Wei County, Laishui County, Fuping County, Tang County, Laiyuan County, Wangdu County, Yi County, Quyang County, Shunping County, Xuanhua District, Wanquan District, Chongli District, Kangbao County, Guyuan County, Shangyi County, Yu County, Yangyuan County, Huai 'an County, Chicheng County, Chengde County, Long Hua County, Fengning Manchu Autonomous County, Weichang Manchu Mongolian Autonomous County, Pingquan City, Yanshan County, Nanpi County, Wuyi County, Wuqiang County, Raoyang County, Fucheng County, Yunzhou District, Yanggao County, Guangling County, Lingqiu County, Hunyuan County, Youyu County, Wutai County, Fanshi County, Wuchuan County, Ar Horqin Banner Banner, Bahrain Left Banner, Linxi County, Wengniute Banner, Harqin Banner, Ningcheng County, Aohan Banner, Khorqin Left Wing Middle Banner, Khorqin Left Wing Rear Banner, Kulun Banner, Naiman Banner, Morin Dawa Daur Autonomous Banner, Oroqen Autonomous Banner, Zhuozi County, Huade County, Shangdu County, Xinghe County, Chahar Right wing Middle banner, Chahar Right wing Rear banner, Siziwang Banner, Arshan City, Khorqin Right Wing Front banner, Khorqin Right Wing Middle banner, Jalaid Banner, Tuquan County, Sonid Right Banner, Taibus Banner, Zhengxiangbai Banner, Jingyu County, Zhenlai County, Tongyu County, Da 'an City, Longjing City, Helong City, Wangqing County, Antu County, Yanshou County, Longjiang County, Tailai County, Gannan County, Fuyu County, Kedong County, Suibin County, Raohe County, Lindian County, Huannan County, Huachuan County, Tangyuan County, Tongjiang City, Fuyuan City, Wangkui County, Lanxi County, Qinggang County, Mingshui County, Sixian County, Fan County, Taiqian County

**2023:**

Lingshou County, Zanhuang County, Daming County, Julu County, Xinhe County, Guangzong County, Pingxiang County, Wei County, Laishui County, Fuping County, Tang County, Laiyuan County, Wangdu County, Yi County, Quyang County, Shunping County, Xuanhua District, Wanquan District, Chongli District, Kangbao County, Guyuan County, Shangyi County, Yu County, Yangyuan County, Huai’an County, Chicheng County, Chengde County, Longhua County, Fengning Manchu Autonomous County, Weichang Manchu Mongolian autonomous County, Pingquan City, Yanshan County, Nanpi County, Wuyi County, Wuqiang County, Raoyang County, Fucheng County, Yunzhou District, Yanggao County, Guangling County, Lingqiu County, Hunyuan County, Youyu County, Wutai County, Fanshi County, Wuchuan County, Ar Horqin Banner, Bahrain Left Banner, Bahrain right flag, Linxi County, Wengniute banner, Harqin Banner, Ningcheng County, Aohan Banner, Khorqin Left Wing Middle Banner, Khorqin Left Wing Rear Banner, Kulun Banner, Naiman Banner, Morin Dawa Daur Autonomous Banner, Oroqen Autonomous Banner, Zhuozi County, Huade County, Shangdu County, Xinghe County, Chahar Right wing Middle banner, Chahar Right wing Rear banner, Siziwang Banner, Arshan City, Khorqin Right Wing Front banner, Khorqin Right Wing Middle banner, Jalaid Banner, Tuquan County, Sonid Right Banner, Taibus Banner, Zhengxiangbai Banner, Jingyu County, Zhenlai County , Tongyu County, Da 'an City, Yanji City, Longjing City, Helong City, Wangqing County, Antu County, Yanshou County, Longjiang County, Tailai County, Gannan County, Fuyu County, Kedong County, Baiquan County, Suibin County, Raohe County, Lindian County, Huanan County, Huachuan County, Tangyuan County, Tongjiang City, Fuyuan City, Wangkui County, Lanxi County, Qinggang County, Mingshui County, Fan County, Taiqian County, Gangcha county
